# Both radiographical and pathological lymph node statuses are independent predictors for survival following neoadjuvant chemotherapy and radical cystectomy for cT3/4 or cN+ bladder cancer

**DOI:** 10.1007/s00345-022-04187-w

**Published:** 2022-10-21

**Authors:** Julia Wagner, Ricarda Simon, Jakob Wolf Büchler, Florian Kirchhoff, Viktoria Kehl, Margitta Retz, Juergen Erich Gschwend, Andreas Sauter, Thomas Horn

**Affiliations:** 1 Department of Urology, School of Medicine, Klinikum Rechts der Isar, Technical University of Munich, Ismaningerstraße 22, 81675 Munich, Germany; 2 Institute for AI and Informatics in Medicine, Technical University of Munich, Munich, Germany; 3 Institute for Diagnostic and Interventional Radiology, Technical University of Munich, Munich, Germany

**Keywords:** Preoperative chemotherapy, Bladder cancer, Lymph node metastasis

## Abstract

**Introduction:**

Urothelial bladder cancer (UBC) with clinical suspicion of locally advanced growth or pelvic lymphogenic spread has a high risk of progression and death.

**Patients and methods:**

Bladder cancer patients with locally advanced (cT3/4) tumor growth or suspected pelvic lymphogenic spread (cN+) were treated with preoperative cisplatin-containing chemotherapy and consolidative cystectomy with pelvic lymphadenectomy. We aimed to identify prognostic factors and describe the patients’ oncological outcome.

**Results:**

A complete dataset including follow-up data was available for 96 patients. In a univariate analysis, we identified cN stage (cN+ vs cN-, HR 2.7, 95% CI 1.3–6.0), response to chemotherapy (HR 0.2, 95% CI 0.1–0.5), ypT stage *(ypT0/is/1 vs ypT2-4,* HR 3.1, 95% CI 1.4–6.8), ypN stage (ypN + vs ypN-, HR 7.9, 95% CI 3.7–17.0), resection status (HR 4.4, 95% CI HR 1.5–13.0) as significantly associated with cancer-specific survival. In a multivariate regression analysis, both cN and ypN statuses were validated as independent prognostic factors for cancer-specific survival (cN: HR 2.6, 95% CI 1.1–6.1; ypN: HR 5.5, 95% CI 2.0–15.1).

**Discussion:**

Lymph node status was identified as a prognostic marker in a high-risk cohort of UBC patients treated with inductive chemotherapy and cystectomy. Establishing cN status as a prognosticator underlines the necessity to aggressively treat these patients despite reported impreciseness of imaging procedures in UCB. Patients with histologically positive lymph nodes following preoperative chemotherapy have a very poor prognosis, and thus, the need for adjuvant systemic treatment is emphasized.

**Conclusion:**

Both clinically and pathologically affected lymph nodes convey a poor prognosis in bladder cancer and necessitate aggressive treatment.

## Introduction

Neoadjuvant cisplatin-containing chemotherapy (NAC) is associated with an overall survival benefit of 5–8% [1, 2] in muscle-invasive urothelial bladder cancer (UBC) and according to international guidelines should be offered to all cisplatin-eligible patients prior to radical cystectomy [3]. Nevertheless, NAC is still not widely accepted for different reasons [4, 5]. One debate is the potential overtreatment of patients with pT2pN0 disease, as cystectomy series show a favorable prognosis for patients with pT2 tumors in comparison with patients with locally advanced pT3/T4 tumors [6]. However, in metastatic UBC cisplatin-based chemotherapy is a widely accepted standard [3, 7].

Clinically regional node-positive bladder cancer with lymphogenic spread within the pelvis is a poorly described entity representing a transition from localized to metastatic disease. It is related to a rather poor prognosis with a reported 5-year survival of 20–60% [6, 8]. Common guidelines do not specifically address the management of these patients, and they have been excluded from seminal clinical trials examining the role of NAC [3, 9].

Staging of bladder cancer patients has high inherent uncertainties with a sensitivity for the detection of lymph node metastases of only 40–60% for both CT and MRI [10–13]. Specificity is reported to be higher but is also compromised by potential inflammatory changes after transurethral resection. Regarding local tumor staging and the differentiation between T3/4 and T2 disease, an accuracy of 80% is reported [14].

Our institution has been using a stringent treatment protocol in the last decade recommending primary systemic treatment with gemcitabine and cisplatin to patients with muscle-invasive UBC and imaging signs for either clinically locally advanced tumor growth (cT3/4) or involvement of pelvic lymph nodes (cN+). As the treatment strategy in these high-risk patients may differ from the neoadjuvant treatment population with cN0 disease, we prefer the term inductive chemotherapy for this patient group instead.

In this manuscript, we present the outcome of this high-risk UBC cohort and identify pelvic lymph node status as the most important prognostic factor.

## Patients and methods

Patients. Patients receiving inductive chemotherapy with gemcitabine and cisplatin and subsequent cystectomy for cT3/4 or cN+ bladder cancer between 2010 and 2021 were retrospectively identified. All cystectomies were done with an open surgical approach. Extended pelvic lymphadenectomy was performed to minimize staging errors [15]. Patients with a primary progression during cisplatin-containing chemotherapy were excluded as well as patients who did not complete at least three cycles of chemotherapy. Imaging studies of all patients were reviewed by one experienced uro-radiologist (AS) regarding local tumor staging and lymph node status. Pelvic lymph nodes with a short-axis diameter of more than 8 mm were considered as metastases. Patients with suspicious retroperitoneal lymph nodes (cM1) were excluded from this analysis as well as patients with distant metastases. Tumoral response to chemotherapy was defined as < ypT2ypN0 in the final histology specimen. The study was approved by the local ethics committee (number 2012/5292). All patients signed written informed consent in compliance with the Declaration of Helsinki.

### Methods

Cancer-specific survival was used for all statistical analyses. Survival estimates were done using the Kaplan–Meier method, and subgroups were compared using the Log-rank test. Univariate and multivariate Cox proportional hazards models were used to investigate the influence of the covariates on the time to cancer-specific death. All tests were performed two-sided with a significance level of 5% without adjusting for multiplicity.

## Results

In total, 96 patients with sufficient follow-up data were included. The 2-year cancer-specific survival estimate was 79.0% (95% CI 70.6–87.4%). The 5-year cancer-specific survival estimate was 64.9% (95% CI 52.7–77.1%). Thirty-one patients have died after a median of 12 months, and the median follow-up time of surviving patients was 40 months. Detailed patient characteristics are shown in Table 1. Regarding initial clinical staging, 43 patients (44.8%) had clinically positive pelvic lymph nodes (cN+), and 81 patients (84.4%) presented with locally advanced tumor growth (cT3/4)). Following inductive chemotherapy and radical cystectomy, a total of 29 patients (30.2%) showed a complete response (ypT0 ypN0) to chemotherapy, and 48 patients (50.0%) showed a response defined as < ypT2 ypN0 in the cystectomy specimen. The median number of removed lymph nodes was 24.

**Table 1:**
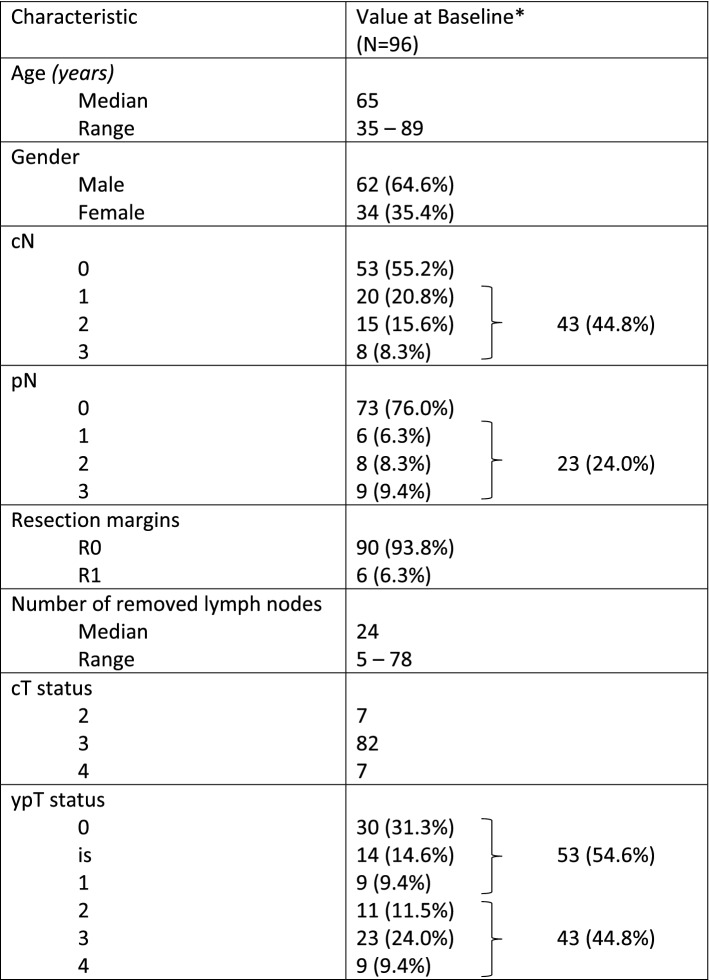
Baseline characteristics of the patients

*Values are summarized with absolute and relative frequencies unless otherwise stated

Of 43 patients with clinically positive lymph nodes, 29 patients (67.4%) had a ypN0 status. Complete response rate (ypT0 ypN0) and response rate (< ypT2 ypN0) in the cN+ group were 10/43 (23.3%) and 17/43 (39.5%), respectively. Focusing on patients with clinically normal lymph node status, 9 of 53 patients (17.0%) had lymph node metastases (ypN+) in the final histologic analysis.

Patients with a ypN+ status had a very poor prognosis, and 17 of 23 ypN+ patients (74%) died after a median time of nine months. The estimated 2-year cancer-specific survival in this subgroup was 35.4% (95% CI 15.0–55.9%).

**Fig. 1:**
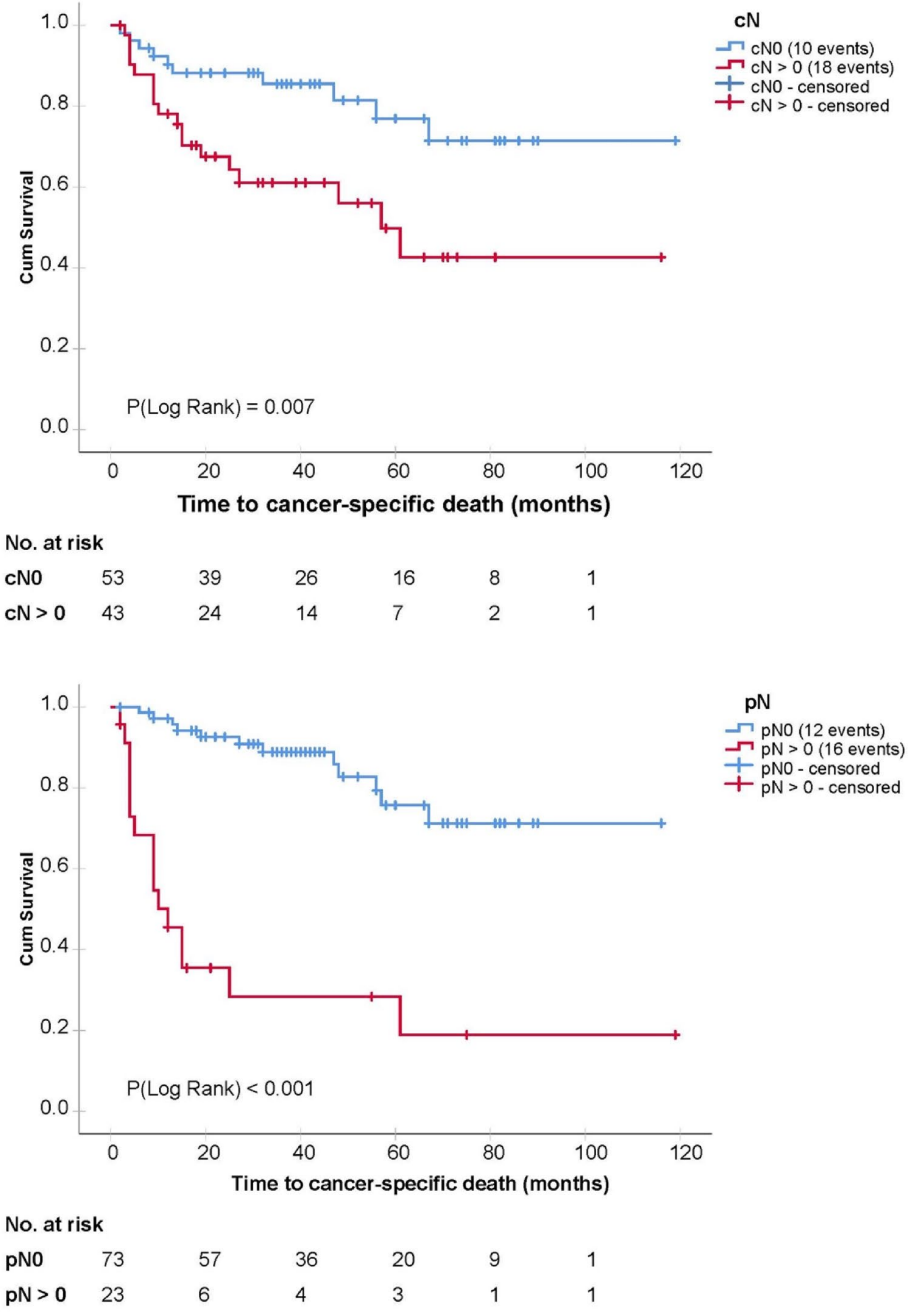
Kaplan–Meier curves depicting cancer-specific survival in dependency of pathological lymph node status after cystectomy (below) and clinical lymph node status before the initiation of chemotherapy (above)

**Fig. 2:**
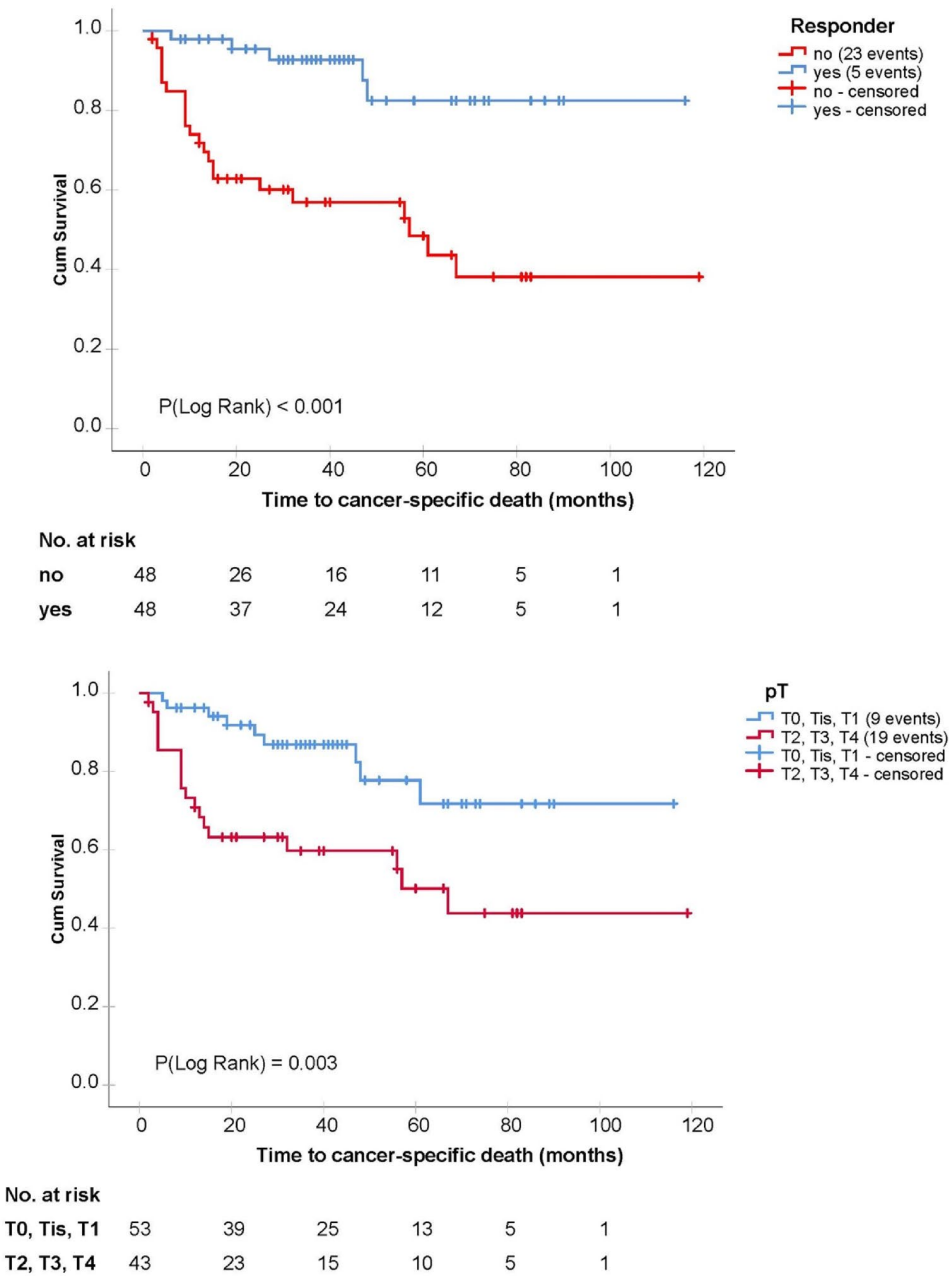
Kaplan–Meier curves of cancer-specific survival in dependency of pathological T stage in the cystectomy specimen (below) and response to chemotherapy defined as a pathological T stage < ypT2ypN0 (above)

A statistically significant association with cancer-specific survival in univariate analyses was found for cN status, ypN status, ypT status, resection margins and response status, whereas age, gender and the number of removed lymph nodes were not significantly associated with cancer-specific survival. Details are shown in Table 2 and corresponding Kaplan–Meier curves in Fig. 1 (cN, ypN) and Fig. 2 (ypT stage, response status).

**Table 2:** Results of the univariate and multivariate Cox proportional hazards regression analysis

Variable	Univariate models			Multivariate model		
	*p* value	HR	(95% CI)	*p* value	HR	(95% CI)
cN positive	0.011	2.7	(1.3, 6.0)	0.029	2.6	(1.1, 6.1)
ypN positive	< 0.001	7.9	(3.7, 17.0)	0.001	5.5	(2.0, 15.1)
Positive response status	< 0.001	0.2	(0.1, 0.5)	0.720	0.7	(0.1, 3.9)
ypT (0–1 vs. 2,3,4)	0.005	3.1	(1.4, 6.8)	0.309	1.9	(0.6, 6.2)
Resection margins (R1 vs. R0)	0.007	4.4	(1.5, 13.0)	0.830	0.9	(0.3, 2.9)

Hazard ratios (HR) for cancer-specific death are shown with their 95% confidence intervals (CI) and corresponding p values of the Wald statistic from the models

*p* values < 0.05 were considered statistically significant

*cN* clinical lymph node status, *pN* pathological lymph node status, *ypT* pathological T stage after chemotherapy and cystectomy,

For statistical analyses, cN and ypN statuses were dichotomized in “positive” and “negative,” as there was no hint for clinically meaningful differences between the subgroups of positive lymph node status cN1, cN2 and cN3 as well as ypN1, ypN2 and ypN3. Furthermore, pathological T stage after cystectomy was dichotomized in < ypT2 and >  = ypT2.

Due to the limited number of patients with a clinically localized stage cT2, we did not perform a formal comparison of these with cT3/4 patients.

Multivariate regression models were calculated and identified both clinical and pathological lymph node statuses as statistically independent prognostic variables for cancer-specific survival (cN status: HR 2.6, 95% CI (1.1, 6.1), *p* = 0.029; ypN status: HR 5.5, 95% CI (2.0, 15.1), *p* = 0.001). Resection margins, response status and ypT stage were not significantly associated with cancer-specific survival in multivariate analyses.

## Discussion

Current guidelines recommend to offer neoadjuvant cisplatin-containing chemotherapy to all patients with muscle-invasive bladder cancer. Nevertheless, neoadjuvant chemotherapy is still not widely accepted due to the risk of potential overtreatment in a subgroup of patients [4, 5]. Selection of patients for neoadjuvant treatment is challenging. An approach to offer inductive chemotherapy to patients with cT3/4 or cN+ tumors and primary cystectomy to those with a cT2 cN0 stage may be a reasonable compromise, but there is sparse published data to support this strategy. Here, we report outcome data for a cohort of bladder cancer patients with either cT3/4 or cN+ stages, treated with preoperative chemotherapy followed by radical cystectomy. As recently published, there is no clinically relevant difference between a robotic surgical approach and open cystectomy [16]. All patients in this study received open cystectomy.

Pathological response to neoadjuvant chemotherapy has been described as a surrogate marker for survival [17, 18]. We observed a pathological complete response (pCR) of 30.2%. Recently, a randomized phase III trial reported a pCR of 36% after 4 cycles of neoadjuvant treatment with gemcitabine and cisplatin in a cohort of patients with muscle-invasive bladder cancer without lymph node metastases [19]. These similar numbers of complete responders underline the high efficacy of inductive chemotherapy. Furthermore, 67% of patients in our study staged cN+ had no signs of pathological lymph node involvement after chemotherapy (ypN0). Focusing on cN+ patients, the largest published cohort with 304 patients displayed a pCR rate of 14.5% and a pN0 rate of 48%. Both rates are higher in our study (pCR 23.3%, pN0 after cN+: 67%) which may reflect improvements in staging modalities and improved patient selection.

We identified cN stage as significantly associated with cancer-specific survival. This underlines the importance of the results of pretreatment imaging studies as treatment decision tools although limitations of both CT and MRI in pelvic lymph node imaging, especially its low sensitivity, are well known [10–13]. Previously, our group reported the prognostic impact of clinical lymph node status prior to radical cystectomy without inductive chemotherapy [20]. The confirmation of these results in this study also for patients receiving inductive chemotherapy underlines the prognostic importance of pretreatment imaging studies.

On the other hand, we have been able to show in a prior study that TURBT prior to imaging studies rarely leads to overstaging [14], which further strengthens the role of initial CT imaging. In locally advanced (pT3/4) disease, oncologic outcomes are reported to be unsatisfying. Also, the risk of occult lymph node metastases in locally advanced disease is substantial in the light of the aforementioned low sensitivity of pelvic imaging for lymph node metastases. This is underlined in our study with 17% of cN0 patients showing ypN+ disease even after at least three cycles of cisplatin-based chemotherapy. This strengthens our hypothesis to advocate inductive chemotherapy also in cT3/4 cN0 patients.

The very poor prognosis of ypN+ patients is a strong argument for aggressive further treatment of these patients. In metastatic bladder, cancer treatment with a PD-1/PD-L1 antibody is recommended for second-line treatment in patients with progression after platinum-based chemotherapy [21]. As ypN+ patients can be regarded as platinum-resistant, an early immunooncological treatment is most likely the best next step. Only recently, nivolumab has been approved for adjuvant treatment for ypT2 or ypN+ patients [22].

We want to emphasize that an extended pelvic lymphadenectomy as performed in this study is necessary to minimize staging errors with the potential consequence of precluding understaged patients from adjuvant treatment.

We were able to confirm a recent study that the response to inductive chemotherapy in bladder cancer is independent from age [23]. Discrepant from another trial, we found no association between the number of removed lymph nodes and cancer-specific survival [24].

The strengths of our study are the stringently used selection criteria for inductive chemotherapy and radiological review of all patients. Limitations are the non-randomized design and monocentric approach.

## Data Availability

The data that support the findings of this study are available from the corresponding author (TH) upon request.
